# Non-high density lipoprotein cholesterol versus low density lipoprotein cholesterol as a discriminating factor for myocardial infarction

**DOI:** 10.1186/1756-0500-5-640

**Published:** 2012-11-17

**Authors:** Manoj Sigdel, Binod Kumar Yadav, Prajwal Gyawali, Prashant Regmi, Sushil Baral, Shyam Raj Regmi, Bharat Jha

**Affiliations:** 1 Department of Biochemistry, Manipal College of Medical Sciences, Pokhara, Nepal; 2 Shahid Gangalal National Heart Centre, Bansbari, Kathmandu, Nepal; 3 Charles Sturt University, New South Wales, 2640, Australia; 4 Yeti Institute of Health Sciences, Kathmandu, Nepal; 5 Department of Biochemistry, Nepal Medical College, Kathmandu, Nepal; 6 Department of Biochemistry, Institute of Medicine, Kathmandu, Nepal

**Keywords:** Dyslipidemia, LDL cholesterol, Myocardial infarction, non-HDL cholesterol

## Abstract

**Background:**

Serum total cholesterol (TC) and LDL cholesterol (LDL-C) have been used as major laboratory measures in clinical practice to assess cardiovascular risk in the general population and disease management as well as prognosis in patients. However, some studies have also reported the use of non-HDL cholesterol (non-HDL-C). As non-HDL-C can be calculated by subtracting HDL-C from TC, both of which do not require fasting blood sample in contrast to LDL-C which requires fasting blood sample, we aimed to compare non-HDL-C with LDL-C as a predictor of myocardial infarction (MI).

**Methods:**

This hospital based cross sectional study was undertaken among 51 cases of MI and equal number of controls. MI was diagnosed based on the clinical history, ECG changes and biochemical parameters. 5 mL of fasting blood sample was collected from each research participant for the analysis of lipid profile. Non-HDL-C was calculated by using the equation; Non-HDL-C = TC – HDL-C. Statistical analysis was performed using SPSS 14.0.

**Results:**

42 MI cases were dyslipidemic in contrast to 20 dyslipidemic subjects under control group. The differences in the median values of each lipid parameter were statistically significant between MI cases and controls. The lipid risk factors most strongly associated with MI were HDL-C (OR 5.85, 95% CI 2.41-14.23, *P* value = 0.000) followed by non-HDL-C (OR 3.77, 95% CI 1.64-8.66, *P* value = 0.002), LDL-C/HDL-C (OR 3.38, 95% CI 1.44-7.89, *P* value = 0.005), TC/HDL-C (OR 2.93, 95% CI 1.36-7.56, *P* value = 0.026), LDL-C (OR 2.70, 95% CI 1.20-6.10, *P* value = 0.017), TC (OR 2.68, 95% CI 1.04-6.97, *P* value = 0.042) and Tg (OR 2.54, 95% CI 1.01-6.39, *P* value = 0.047). Area under the receiver operating curve was greater for non-HDL-C than for LDL-C. Non-HDL-C was also found to be more sensitive and specific than LDL-C for MI.

**Conclusions:**

HDL-C and non-HDL-C are better discriminating parameters than LDL-C for MI. Thus, we can simply perform test for HDL-C and non-HDL-C both of which do not require fasting blood sample rather than waiting for fasting blood sample to measure LDL-C.

## Background

A dramatic increase in the incidence of myocardial infarction (MI) has been observed in many countries. It has become one of the major causes of morbidity and mortality in the middle aged and elderly population. Generally, MI results from risk factors for atherosclerosis [1]. Dyslipidemia has been proven to be an important modifiable risk factor for MI [2]. Serum total cholesterol (TC) and low density lipoprotein cholesterol (LDL-C) have been used as major laboratory measures in clinical practice to assess cardiovascular risk in the general population and disease management as well as prognosis in patients [3]. Recent studies, however, have shown that non-high density lipoprotein cholesterol (non-HDL-C) concentration is similar to or better than LDL-C alone in the prediction of cardiovascular disease (CVD) incidence and mortality [4-7]. Unlike LDL-C, which can be incorrectly calculated in the presence of postprandial hypertriglyceridemia, non-HDL-C is reliable when measured in the non-fasting state [8]. Non-HDL-C can be calculated by subtracting HDL-C from TC both of which do not require fasting blood sample. Therefore, we aimed to compare non-HDL-C with LDL-C as a predictor of MI.

## Methods

This is a cross-sectional study among 51 cases of MI admitted at Coronary care unit of Shahid Gangalal National Heart Centre, Bansbari, Kathmandu from 25th July to 24th Nov, 2010. MI was diagnosed based on the clinical history, ECG changes and biochemical parameters. During the time of admission of the patient, Troponin I was performed by Acon kit and CK-MB was performed by an analyser from Dade Baring. Fasting blood samples for lipid profile were collected on the morning of second day of admission. Equal number of age and sex matched healthy controls were chosen for comparison. The control subjects were such chosen that they did not have a history of CVD. Both patients and controls were living in the same area during the time period of the study. Lipid profile: TC, triglycerides (Tg) and HDL-C were performed in the biochemistry laboratory of Tribhuvan University Teaching Hospital, Kathmandu, from fully automated biochemistry analyzer BT 2000 plus. Non-HDL-C was calculated as TC minus HDL-C. LDL-C was calculated using Friedwald’s equation; LDL-C = TC – (HDL-C + Tg/5). LDL-C/HDL-C and TC/HDL-C ratios were calculated mathematically. Research proposal was approved by Institutional Review Board, Institute of Medicine and written consent was taken from all the participants. National Cholesterol Education Programme Adult Treatment Panel III (NCEP-ATPIII) guidelines was referred to define dyslipidemia [3]. According to NCEP-ATPIII guideline, hypercholesterolemia is defined as TC > 200 mg/dl, LDL-C as >100 mg/dl, hypertriglyceridemia as Tg >150 mg/dl and low HDL-C as <40 mg/dl. Dyslipidemia was defined by the presence of one or more than one abnormal serum lipid concentration. Non-HDL-C was considered normal below 130 mg/dl. Statistical analysis was performed using SPSS 14. Test for normality of data was done by Shapiro-Wilk Test. As there was not normal distribution of data, medians were compared by using Mann–Whitney U test and correlations among the different lipid parameters were determined using Spearman’s rank correlation test. Sensitivity, specificity, positive predictive value (PPV) and negative predictive value (NPV) of each lipid parameters were calculated by using chi-square test. The odds ratio (OR) (and the corresponding confidence interval) of having MI was estimated using Mantel-Haenszel common odds ratio. Receiver Operating Characteristic (ROC) curve was constructed and area under the curve (AUC) was obtained for lipid parameters. All reported probability values (*P*-values) were based on two-sided tests and *P* value <0.05 was considered statistically significant.

## Results

Among the total of 51 MI diagnosed cases, 36 (70.6%) were males and 15 (29.4%) were females. The mean age of MI cases was 60.96 years; ranging from 30 years to 98 years. Dyslipidemia was found on 42 (82.35%) cases. Out of four sub-sets of lipid profile, namely: TC, LDL-C, HDL-C and Tg, nine cases were dyslipidemic by one parameter, twenty cases by two parameters, nine cases by three parameters, and four cases by all four parameters. Hypercholesterolaemia was found on 17 (33.34%), hypertriglyceridaemia on 18 (35.29%), and an increase of LDL-C was found on 27 (57.44%) cases. Decreased HDL-C level was found on 30 (58.82%) cases (Table 1). However, only 20 (39.21%) of the controls were found to be dyslipidemic by one or more parameters. For each lipid parameter, the differences in median lipid values were statistically significant between MI cases and controls (Table 2). The lipid risk factors most strongly associated with MI were HDL-C (OR 5.85, 95% CI 2.41-14.23, *P* value = 0.000) followed by non-HDL-C (OR 3.77, 95% CI 1.64-8.66, *P* value = 0.002), LDL-C/HDL-C (OR 3.38, 95% CI 1.44-7.89, *P* value = 0.005), TC/HDL-C (OR 2.93, 95% CI 1.36-7.56, *P* value = 0.026), LDL-C (OR 2.70, 95% CI 1.20-6.10, *P* value = 0.017), TC (OR 2.68, 95% CI 1.04-6.97, *P* value = 0.042) and Tg (OR 2.54, 95% CI 1.01-6.39, *P* value = 0.047). These findings were confirmed in the ROC analysis, which also suggested a strong association of these variables with MI. AUC was highest for HDL-C (0.834) followed by TC/HDL-C (0.789), LDL-C/ HDL-C (0.748), non-HDL-C (0.734), TC (0.709), LDL-C (0.708), and Tg (0.638). The diagnostic value of these lipid parameters in MI at cut-off points suggested by NCEP ATP III is given in Table 3. Non-HDL-C was significantly correlated with TC (r = 0.991, *P* < 0.0001), LDL-C (r = 0.960, *P* < 0.0001), TC/HDL-C (r = 0.927, *P* < 0.0001), LDL-C/HDL-C (r = 0.915, *P* < 0.0001), HDL (r = −0.445, *P* < .0001) and Tg (r = 0.410, *P* < 0.0001). However, the correlation of LDL was insignificant with Tg (r = 0.152, P > 0.05).

**Table 1:** Lipid profile status of MI cases

**Total cholesterol**		**Triglycerides**		**HDL-cholesterol**		**LDL-cholesterol**		**Non-HDL cholesterol**	
**Normal**	**High**	**Normal**	**High**	**Normal**	**Low**	**Normal**	**High**	**Normal**	**High**
34	17	33	18	21	30	24	27	30	21
(66.66%)	(33.34%)	(64.71%)	(35.29%)	(41.18%)	(58.82%)	(42.56%)	(57.44%)	(58.52%)	(41.17%)

**Table 2:** Comparison of lipid status between MI cases and controls (median, 25th-75th percentile)

**Parameters**	**MI cases**	**Control**	***P *value**
TC (mg/dl)	173.70	139.32	0.000
	142.82-212.30	119.97-174.15	
Tg (mg/dl)	131.55	113.94	0.016
	96.47-192.94	95.00-138.00	
LDL-C (mg/dl)	108.08	69.15	0.000
	73.34-146.68	48.86-105.65	
HDL-C (mg/dl)	38.60	46.44	0.000
	34.74-42.46	42.57-50.31	
non-HDL-C (mg/dl)	135.10	96.75	0.000
	100.36-173.70	69.66-131.58	
LDL-C/ HDL-C	2.80	1.45	0.000
	1.82-3.50	1.01-2.45	
TC/ HDL-C	4.67	3.07	0.000
	3.70-5.00	2.46-3.89	

Legend: TC- Total cholesterol; Tg- Triglycerides ; LDL-C- Low density lipoprotein cholesterol; HDL-C- High density lipoprotein cholesterol; non-HDL-C- Non-high density lipoprotein cholesterol; LDL-C/HDL-C- Ratio of LDL-C to HDL-C ; TC/HDL-C- Ratio of TC to HDL-C.

**Table 3:** Diagnostic value of lipid parameters in MI

**Parameters**	**Cut-off values**	**Sensitivity (%)**	**Specificity (%)**	**PPV (%)**	**NPV (%)**
TC	200 mg/dl	33.33	84.31	68.0	55.84
Tg	150 mg/dl	35.29	82.35	66.67	56.0
LDL-C	100 mg/dl	52.94	70.59	64.29	60
HDL-C	40 mg/dl	58.82	80.39	75.0	66.13
non-HDL-C	130 mg/dl	58.82	72.55	68.18	63.79
LDL-C/HDL-C	2.5	50.98	76.47	68.42	60.94
TC/ HDL-C	5	35.29	84.31	69.23	56.58

**Legend:** TC- Total cholesterol; Tg- Triglycerides ; LDL-C- Low density lipoprotein cholesterol; HDL-C- High density lipoprotein cholesterol; non-HDL-C- Non-high density lipoprotein cholesterol; LDL-C/HDL-C- Ratio of LDL-C to HDL-C ; TC/HDL-C- Ratio of TC to HDL-C; PPV- Positive predictive value; NPV- Negative predictive value.

## Discussion

This is probably the first case–control study in our part of the world aiming at identifying the importance of non-HDL-C in patients with MI. In our study, we identified HDL-C as the lipid parameter most strongly associated with MI followed by non-HDL-C. The association between low levels of HDL-C and an increased risk for CVD has been well established through epidemiological and clinical studies [9]. In a prospective study conducted among 1,799 Finnish men, it was shown that serum HDL-C of less than 42 mg/dl was associated with a 3.3-fold risk of MI. Adjustments for body mass index (BMI), history of diabetes mellitus (DM) (yes versus no), serum Tg and LDL-C concentration did not influence this relative hazards. Also, the study showed that serum LDL-C, Tg, fasting blood glucose level, BMI or history of DM was not significantly associated with the risk of MI [10]. The Framingham heart study also showed that low level of HDL-C was the major potent lipid risk factor for the incidence of coronary heart diseases [11]. These findings are supported by the potential antiatherogenic properties of HDL-C, including its mediation of reverse cholesterol transport, in which cholesterol from peripheral tissues is returned to the liver for excretion in the bile [12]. Moreover, HDL-C inhibits Ca^2+^ induced procoagulant activity on erythrocyte membranes [13]. HDL-C has also been shown to promote fibrinolysis [14]. Anti-oxidative property of HDL-C could be the other cardio-protective mechanism [15].

The differences in the median values of each lipid parameter were statistically significant between MI cases and controls which show that dyslipidemia is one of the factors responsible for the causation of MI. We found a good correlation of non-HDL-C with total as well as LDL cholesterol. This shows that the value of non-HDL cholesterol also reflects the value of total cholesterol as well as LDL cholesterol. Non-HDL cholesterol is simply an estimate of all the atherogenic lipid particles as it includes LDL cholesterol, intermediate density lipoprotein cholesterol as well as very low density lipoprotein cholesterol. Sensitivity and specificity as well as PPV and NPV in the study showed that non-HDL-C is more powerful discriminating factor for MI than LDL-C.

**Figure 1:**
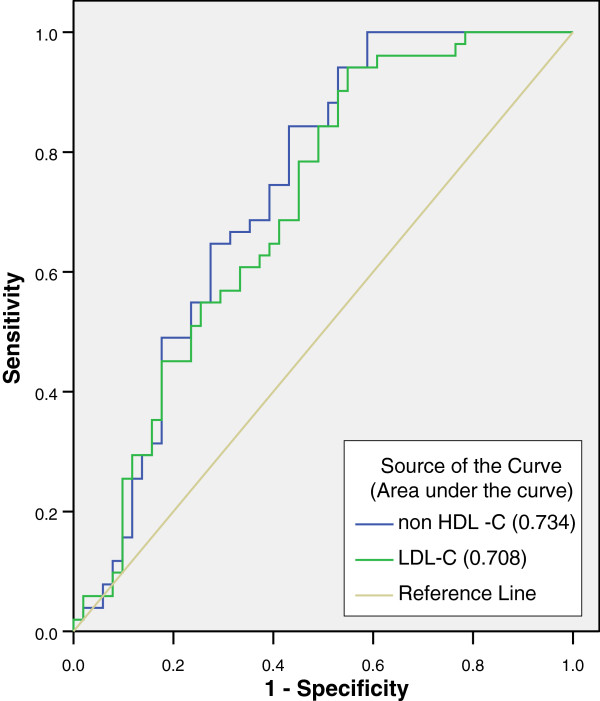
**Receiver operating characteristic curve for non-HDL cholesterol and LDL cholesterol.** Area under the curve is greater for non-HDL cholesterol than for LDL cholesterol which shows that non- HDL cholesterol is better discriminating factor for myocardial infarction than LDL cholesterol.

Similarly, results from the ROC curve also confirm that non-HDL-C is better associated with MI than LDL-C (Figure 1). We also found that non-HDL-C was significantly correlated with all other lipid parameters; however LDL cholesterol was not significantly associated with Tg. The superiority of non-HDL-C over LDL-C may be due to the fact that triglycerides and subsequently triglyceride-rich lipoproteins may play an important role in the causation of MI. 33 cases with MI have higher Tg value in our study. Many studies have shown the involvement of Tg and particularly Tg rich lipoproteins in the pathogenesis of CVD. Tg rich lipoproteins have been shown to induce endothelial dysfunction, enhance monocyte adhesion [16], enter atherosclerotic plaques [17,18] and inhibit reverse cholesterol transport [19]. The peroxidative products within the lipid core is also found to involve in plaque fissuring and lesion disruption [20]. Non-HDL-C has been proposed as a good estimator of the atherogenic potential in patients with high Tg [21]. The findings of Cui et al.; has also demonstrated non-HDL-C as a better predictor of CVD mortality than LDL-C during an average follow up of 19 years in 4462 dyslipidemic patients [4]. The strong association of lipid parameters with MI in our study is in agreement with the study of Goliasch et al.; [22]. Among 102 MI patients recruited by the authors, non-HDL-C was most strongly associated with MI followed by LDL-C. Similarly, Ridker et al.; showed that non-HDL-C, TC/HDL-C and LDL-C/HDL-C were better lipid parameters than LDL-C as a predictor of future cardiovascular events in women [23]. 

Moreover, realizing the atherogenic potential of some Tg rich lipoproteins, NCEP ATP III has also introduced non-HDL-C as a secondary target of treatment in patients with high Tg after achieving LDL-C targets [3]. Non-HDL-C has also several practical advantages in clinical practice. It can be calculated in the non-fasting state by subtracting HDL-C from TC. In addition, it can also be calculated in the setting of hypertriglyceridemia where LDL-C estimation with Friedwald's formula is less accurate and is considered inapplicable in cases where Tg > 400 mg/dl [24].

In our study, we were interested to compare the bad cholesterols; non-HDL-C versus LDL-C as a discriminating factor for MI, and we found that non-HDL-C is a better discriminating parameter than LDL-C for MI. To measure HDL-C or non-HDL-C we do not need fasting blood sample whereas to measure LDL-C fasting blood sample is needed. Thus, the study gives the idea that both HDL-C and non-HDL-C are better discriminating parameters than LDL-C for MI and hence we can analyse random blood for HDL-C and non-HDL-C rather than waiting for fasting sample to measure LDL-C.

## Conclusions

Dyslipidemia is one of the risk factors of MI. HDL-C and non-HDL-C are better discriminating parameters than LDL-C for MI. This study gives the idea that we can simply perform test from random blood sample to measure HDL-C and non-HDL-C rather than waiting for fasting sample to measure LDL-C.

## Abbreviations

AUCM: Area under the curve; BMI: Body mass index; CCU: Coronary care unit; CVD: Cardiovascular diseases; DM: Diabetes mellitus; HDL: High density lipoprotein cholesterol; LDL-C: Low density lipoprotein cholesterol; MI: Myocardial infarction; NCEP-ATPIII: National Cholesterol Education Programme Adult Treatment Panel III; non-HDL-C: Non-high density lipoprotein cholesterol; NPV: Negative predictive value; OR: Odds ratio; PPV: Positive predictive value; ROC: Receiver operating characteristic; TC: Total cholesterol; Tg: Triglycerides.

## Competing interests

The authors declare that they have no competing interests.

## Authors’ contributions

MS drafted the manuscript and performed the interpretation of the data. BKY conceived the study and involved in sample collection and analysis. PG designed the study and drafted the manuscript. PR and SB involved in sample collection and analysis. SRR and BJ participated in the design of the study and revising the manuscript for important intellectual content. All authors read and approved the final manuscript.

## Authors' information

1). Lecturer 2). Medical Lab Technologist 3). phd fellow 4). Lecturer 5). Lecturer 6). Cardiologist 7). Professor and Head, Department of Biochemistry

## References

[B1] Bitla AR Pallavi M Vanaja V Suchitra MM Reddy VS Reddy EP Rao PVLNS Acute Myocardial Infarction in a Southeast Indian Population: Comparison of Traditional and Novel Cardiovascular Risk Factors Res J Med Med Sci 2009 4 2 202 206

[B2] Arnold VE Is there a need for novel cardiovascular risk factors ? Nephrol Dial Transplant 2004 19 761 765 10.1093/ndt/gfh111 15031325

[B3] Expert Panel on Detection Evaluation, and Treatment of High Blood Cholesterol in Adults Executive summary of the third report of the National Cholesterol Education Program (NCEP) Expert Panel on Detection, Evaluation, and Treatment of High Blood Cholesterol in Adults (Adult Treatment Panel III) JAMA 2001 285 2486 2497 10.1001/jama.285.19.2486 11368702

[B4] Cui Y Blumenthal RS Flaws JA Whiteman MK Langenberg P Bachorik PS Bush TL Non-high-density lipoprotein cholesterol level as a predictor of cardiovascular disease mortality Arch Intern Med 2001 161 1413 1419 10.1001/archinte.161.11.1413 11386890

[B5] Farwell WR Sesso HD Buring JE Gaziano JM Non-high-density lipoprotein cholesterol versus low-density lipoprotein cholesterol as a risk factor for a first nonfatal myocardial infarction Am J Cardiol 2005 96 1129 1134 10.1016/j.amjcard.2005.06.044 16214451

[B6] Liu J Sempos CT Donahue RP Dorn J Trevisan M Grundy SM Non-high density lipoprotein and very-low-density lipoprotein cholesterol and their risk predictive values in coronary heart disease Am J Cardiol 2006 98 1363 1368 10.1016/j.amjcard.2006.06.032 17134630

[B7] Ingelsson E Schaefer EJ Contois JH McNamara JR Sullivan L Keyes MJ Pencina MJ Schoonmaker C Wilson PWF D'Agostino RB Vasan RS Clinical utility of different lipid measures for prediction of coronary heart disease in men and women JAMA 2007 298 776 785 10.1001/jama.298.7.776 17699011

[B8] The Expert Panel Third report of the National Cholesterol Education Program (NCEP) Expert Panel on Detection, Evaluation, and Treatment of High Blood Cholesterol in Adults (Adult Treatment Panel III): final report Circulation 2002 106 3143 3421 12485966

[B9] Gordon DH Rifkind BM High-density lipoprotein: the clinical implications of recent studies N Engl J Med 1989 321 1311 1316 10.1056/NEJM198911093211907 2677733

[B10] Salonen JT Salonen R Seppänen K Rauramaa R Tuomilehto J HDL1, HDL2, and HDL3 subfractions, and the risk of acute myocardial infarction. A prospective population study in eastern Finnish men Circulation 1991 84 129 139 10.1161/01.CIR.84.1.129 2060089

[B11] Gordon T Castelli WP Hjortland MC Kannel WB Dawber TR High density lipoprotein as a protective factor against coronary heart disease. The Framingham study Am J Med 1997 62 5 707 714 193398 10.1016/0002-9343(77)90874-9

[B12] Barter P CETP and atherosclerosis Arterioscler Thromb Vasc Biol 2000 20 2029 2031 10.1161/01.ATV.20.9.2029 10978244

[B13] Epand RM Stafford A Leon B Lock PE Tytler EM Segrest JP Anantharamaiah GM HDL and apolipoprotein A-I protect erythrocytes against the generation of procoagulant activity Arterioscler Thromb Vasc Biol 1994 14 11 1775 1783 10.1161/01.ATV.14.11.1775 7947603

[B14] Saku K Ahmad M Glas-Greenwalt P Kashyap ML Activation of fibrinolysis by apolipoproteins of high density lipoproteins in man Thromb Res 1985 39 1 8 10.1016/0049-3848(85)90116-1 4035643

[B15] Boisfer E Stengel D Pastier D Laplaud PM Dousset N Ninio E Kalopissis AD Antioxidant properties of HDL in transgenic mice overexpressing human apolipoprotein A-II J Lipid Res 2002 43 5 732 741 11971944

[B16] Carantoni M Abbasi F Chu L Chen YDI Reaven GM Taso PS Varasteh B Cooke JP Adherence of mononuclear cells to endothelium in vitro is increased in patients with NIDDM Diabetes Care 1997 20 1462 1465 10.2337/diacare.20.9.1462 9283798

[B17] Bates SR Murphy PL Feng Z Kanazawa T Getz GS Very low density lipoproteins promote triglyceride accumulation in macrophages Arteriosclerosis 1984 4 103 114 10.1161/01.ATV.4.2.103 6704048

[B18] Rapp JH Lespine A Hamilton RL Colyvas N Chaumeton AH Tweede-Hardmann J Kotite L Kunitake ST Havel RJ Kane JP Triglyceride-rich lipoproteins isolated by selected-affinity anti-apolipoprotein B immunoabsorption from human atherosclerotic plaque Arterioscler Thromb Vasc Biol 1994 14 1767 1774 10.1161/01.ATV.14.11.1767 7947602

[B19] Palmer AM Murphy N Graham A Triglyceride-rich lipoproteins inhibit cholesterol efflux to apolipoprotein (apo) A1 from human macrophage foam cells Atherosclerosis 2004 173 27 38 10.1016/j.atherosclerosis.2003.12.001 15177121

[B20] Hodis HN Mack WJ Azen SP Alaupovic P Pogoda JM LaBree L Hemphill LC Kramsch DM Blankenhorn DH Triglyceride and cholesterol rich lipoproteins have a differential effect on mild/moderate and severe lesion progression as assessed by quantitative coronary angiography in a controlled trial of lovastatin Circulation 1994 90 42 49 10.1161/01.CIR.90.1.42 8026027

[B21] Grundy SM Low-density lipoprotein, non-high-density lipoprotein, and apolipoprotein B as targets of lipid-lowering therapy Circulation 2002 106 2526 2529 10.1161/01.CIR.0000038419.53000.D6 12427645

[B22] Goliasch G Oravec S Blessberger H Dostal E Hoke M Wojta J Schillinger M Huber K Maurer G Wiesbauer Relative importance of different lipid risk factors for the development of myocardial infarction at a very young age (≤ 40 years of age) Eur J Clin Invest 2012 42 6 631 636 10.1111/j.1365-2362.2011.02629.x 22150092

[B23] Ridker PM Rifai N Cook NR Bradwin G Buring JE Non–HDL Cholesterol, Apolipoproteins A-I and B100, Standard Lipid Measures, Lipid Ratios, and CRP as Risk Factors for Cardiovascular Disease in Women JAMA 2005 294 3 326 333 10.1001/jama.294.3.326 16030277

[B24] Frost PH Havel RJ Rationale for use of non-high-density lipoprotein cholesterol rather than low-density lipoprotein cholesterol as a tool for lipoprotein cholesterol screening and assessment of risk and therapy Am J Cardiol 1998 81 4A 26B 31B 10.1016/s0002-9149(98)00034-4 9526810

